# A Case of Meningovascular Neurosyphilis with Black-Blood MRI Sequences

**DOI:** 10.5334/jbsr.3662

**Published:** 2024-08-14

**Authors:** Brecht Van Berkel, Vincent Sneyers, Sofie Van Cauter

**Affiliations:** 1 University Hospitals of Leuven, Herestraat 49, 3000 Leuven, Belgium; 2 University Hospitals of Leuven, Herestraat 49, 3000 Leuven, Belgium; 3 Ziekenhuis-Oost Limburg, Synaps Park 1, 3600 Genk, Belgium

**Keywords:** Neurosyphilis, MRI, black-blood, stroke

## Abstract

*Teaching point:* Meningovascular neurosyphilis is a cause of stroke in the young, and the diagnosis could be aided by black-blood MRI sequences.

## Case History

A 32-year-old male patient was referred to our hospital with vertigo and dysarthria. An MRI of the brain was performed, which showed multiple areas of diffusion restriction (Figures 1a and 1b, yellow arrowheads), corresponding with acute ischemia. The scanning protocol of this MRI included 3D T1 black-blood-sequences. Black-blood sequences showed multiple areas of vessel wall contrast enhancement, with circumferential enhancement of the basilar artery (Figure 2a, yellow arrowhead) and focal endoluminal contrast enhancement of the V4 segment of the right vertebral artery (Figures 2b and 2c, yellow arrowheads). Furthermore, there were multiple other zones of focal contrast enhancement of both superior cerebellar arteries and the P2/P3 segment of the left posterior cerebral artery (PCA). Time of flight angiography showed a long segmented severe stenosis of the basilar artery, the right vertebral artery and the proximal PCA (Figure 3, yellow arrowheads). An extensive laboratory investigation was performed, including blood tests, CSF tests and urine tests. The CSF tests showed T. pallidum infection, in keeping with syphilis. The final diagnosis was the meningovascular variant of neurosyphilis (NS).

**Figure 1a:**
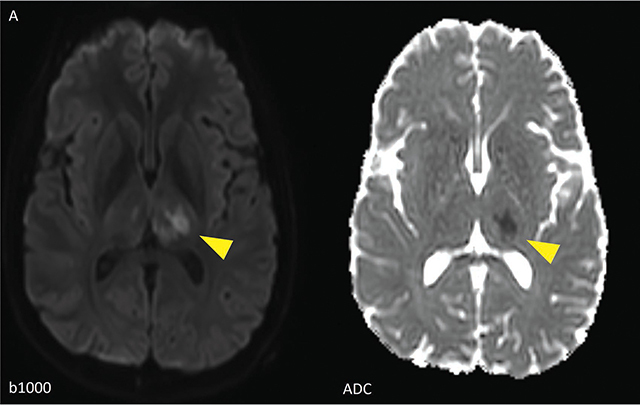
DWI images showing multiple areas of diffusion restriction, corresponding with acute ischemia (yellow arrowheads) in the left thalamus.

**Figure 1b:**
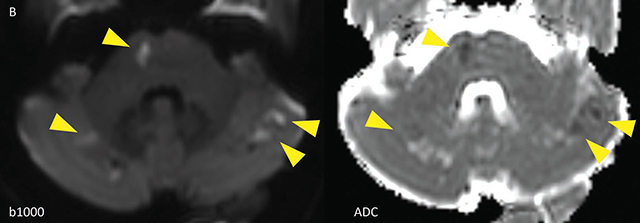
DWI images showing multiple areas of diffusion restriction, corresponding with acute ischemia (yellow arrowheads) in the cerebellum and pons.

**Figure 2a:**
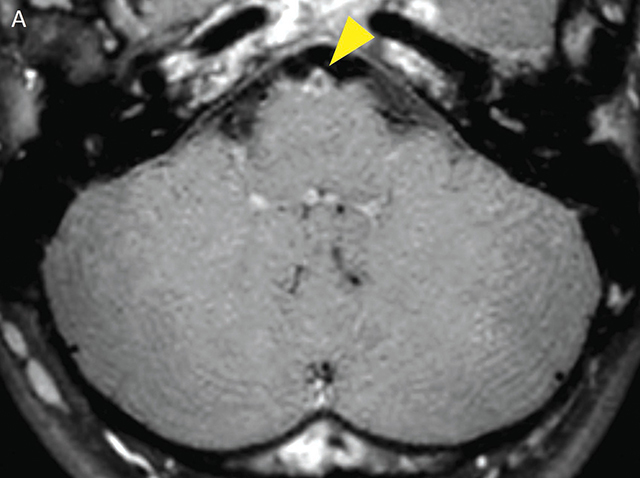
Black-blood-MRI showing eccentric narrowing of the proximal basilar artery and circumferential contrast enhancement in the axial plane (yellow arrowhead).

**Figure 2b:**
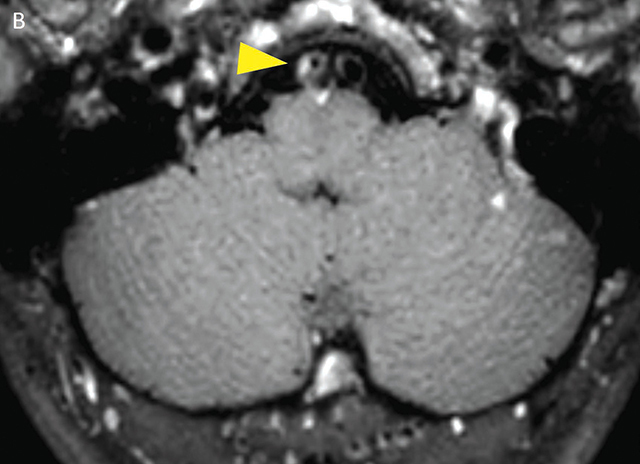
Black-blood-MRI showing focal intraluminal contrast enhancement of the V4 segment of the right vertebral artery in the axial plane (yellow arrowhead).

**Figure 2c:**
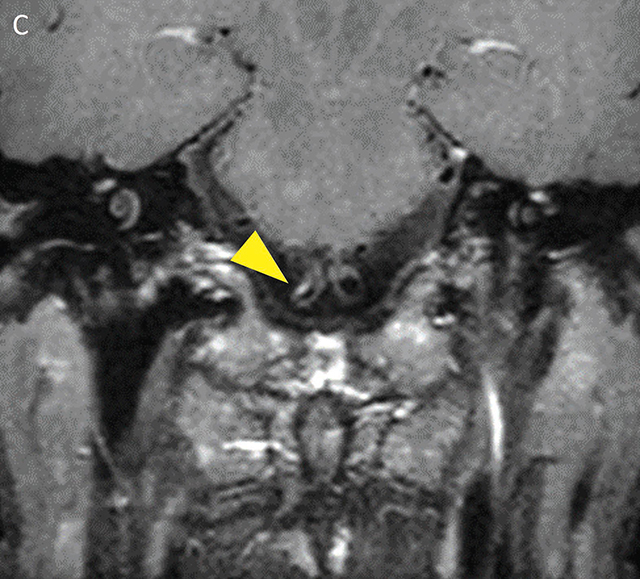
Black-blood-MRI showing focal intraluminal contrast enhancement of the V4 segment of the right vertebral artery in the coronal plan (yellow arrowhead).

**Figure 3:**
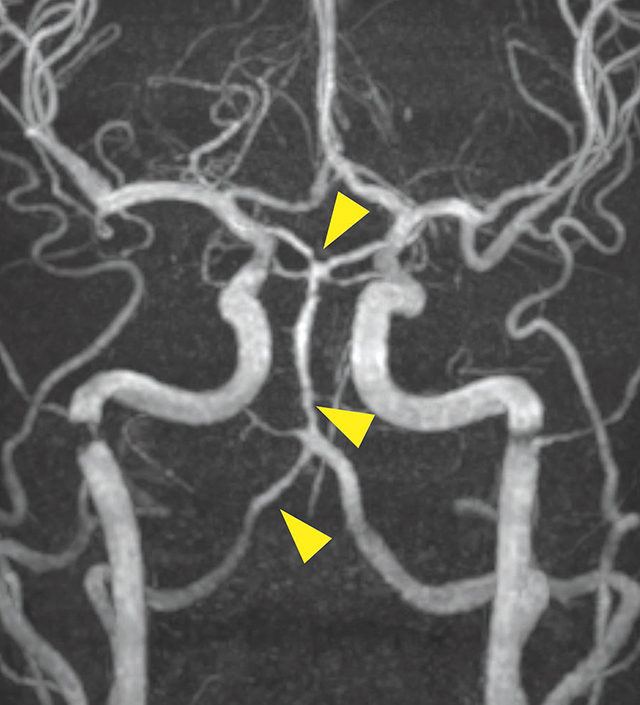
Time of flight angiography showed a long segmented severe stenosis of the basilar artery, the right vertebral artery and the proximal PCA (yellow arrowheads).

## Discussion

NS is an infection of the central nervous system that has a variety of clinical symptoms and imaging findings. Neuroimaging plays an important part in the diagnosis and management of NS. Nevertheless, the imaging features can mimic those of other neurological disorders. Imaging features can also vary according to the clinical manifestations. Nagappa et al. [1] described three forms of NS: neuropsychiatric NS, meningovascular NS and myelopathic NS. The most common imaging feature of neuropsychiatric NS is cortical atrophy, with a predominant involvement of the frontotemporal lobes. For meningovascular NS, the most common imaging features are vascular occlusion and cerebral infarcts. Finally, myelopathic NS has T2W hyperintense signal changes of the spinal cord as the most common imaging feature. (1) The meningovascular form of NS affects large- and medium-sized intracranial vessels, with intimal proliferation and inflammation of the adventitia leading to vascular occlusion, which leads to ischemia and infarction; this vessel involvement is also named Heubner’s arteritis. Black-blood MRI has been used to visualize and analyze the lumen and the outer-wall boundaries of extracranial and intracranial arteries by suppressing signals from the arterial lumen. Black-blood MRI is an excellent technique to evaluate the arterial wall and inflammatory activity and can be used to aid in the diagnosis of meningovascular neurosyphilis. Young patients with an ischemic stroke require an MRI with black-blood images, as vasculitis is a frequent cause of ischemic stroke in this population.

## References

[r1] Nagappa M, Sinha S, Taly AB, Rao SL, Nagarathna S, Bindu PS, et al. Neurosyphilis: MRI features and their phenotypic correlation in a cohort of 35 patients from a tertiary care university hospital. Neuroradiology. 2013;55(4):379–388. 10.1007/s00234-012-1017-9 23274762

